# Determinants of isoniazid preventive therapy completion among people living with HIV attending care and treatment clinics from 2013 to 2017 in Dar es Salaam Region, Tanzania. A cross-sectional analytical study

**DOI:** 10.1186/s12879-020-04997-6

**Published:** 2020-04-10

**Authors:** Masanja Robert, Jim Todd, Bernard J. Ngowi, Sia E. Msuya, Angella Ramadhani, Veryhel Sambu, Isaya Jerry, Martin R. Mujuni, Michael J. Mahande, James S. Ngocho, Werner Maokola

**Affiliations:** 1 Department of Epidemiology and Biostatistics, Institute of Public Health Kilimanjaro Christian Medical University College (KCMUCo), P.O.Box 2240, Kilimanjaro, Tanzania; 2 Mwenge Catholic University (MWECAU), P.O.Box 1226, Moshi, Tanzania; 3 London School of Hygiene and Tropical Medicine (LSTM), London, UK; 4 National Institute for Medical Research-Muhimbili Medical Research Centre, P.O.Box 3436, Dar es Salaam, Tanzania; 5 University of Dar es Salaam College of Health and Allied Sciences, P.O.Box 68, Mbeya, Tanzania; 6 Ministry of Health, Community Development, Gender, Elderly and Children (NACP), Dodoma, Tanzania

**Keywords:** IPT completion, HIV, Tuberculosis, Predictors, Tanzania

## Abstract

**Background:**

Tuberculosis (TB) disease is a common opportunistic infection among people living with HIV (PLHIV). WHO recommends at least 6 months of isoniazid Preventive Therapy (IPT) to reduce the risk of active TB. It is important to monitor the six-month IPT completion since a suboptimal dose may not protect PLHIV from TB infection. This study determined the six-month IPT completion and factors associated with six-month IPT completion among PLHIV aged 15 years or more in Dar es Salaam region, Tanzania.

**Methods:**

Secondary analysis of routine data from PLHIV attending 58 care and treatment clinics in Dar es Salaam region was used. PLHIV, aged 15 years and above, who screened negative for TB symptoms and initiated IPT from January, 2013 to June, 2017 were recruited. Modified Poisson regression with robust standard errors was used to estimate prevalence ratios (PR) and 95% confidence interval (CI) for factors associated with IPT completion. Multilevel analysis was used to account for health facility random effects in order to estimate adjusted PR (APR) for factors associated with IPT six-month completion.

**Results:**

A total of 29,382 PLHIV were initiated IPT, with 21,808 (74%) female. Overall 17,092 (58%) six-month IPT completion, increasing from 42% (773/1857) in year 2013 to 76% (2929/3856) in 2017. Multilevel multivariable model accounting for health facilities as clusters, showed PLHIV who were not on ART had 46% lower IPT completion compared to those were on ART (APR: 0.54: 95%CI: 0.45–0.64). There was 37% lower IPT completion among PLHIV who transferred from another clinic (APR: 0.63: 95% CI (0.54–0.74) compared to those who did not transfer. PLHIV aged 25–34 years had a 6% lower prevalence of IPT completion as compared to those aged 15 to 24 years (APR:0.94 95%CI:0.89–0.98).

**Conclusion:**

The IPT completion rate in PLHIV increased over time, but there was lower IPT completion in PLHIV who transferred from other clinics, who were aged 25 to 34 years and those not on ART. Interventions to support IPT in these groups are urgently needed.

## Background

Tuberculosis (TB) disease is common among people living with HIV (PLHIV), with the lifetime risk of acquiring disease 20–37 times higher compared to people who are HIV negative [1–5]. Tuberculosis co-infection increases the consequences of the HIV burden and contributes to 30–40% of deaths among PLHIV in high endemic regions [6–9]. Globally TB/HIV co-infection is a major public health concern with more than1 million PLHIV co-infected with TB in the year 2017 [7, 10], and 78% of those conifected with TB living in Africa [7]. In Tanzania there were 142,000 new TB cases in 2018, and 34% of these TB cases were PLHIV [11–14]. In Tanzania more than 1 million PLHIV attend care and treatment clinic, and in 96% of all clinic visits they are screened for TB [15]. The consequences of TB/HIV co-infection is an increase in mortality among PLHIV, with the worldwide estimate of 300,000 deaths among PLHIV co-infected with TB in 2013 and 400,000 in 2017 [3, 7, 8, 11–14, 16].

Isoniazid is the common name for isonicotinylhydrazide (INH) and is one of the drugs used for first line treatment for TB infection [17]. Among PLHIV, INH is also used to prevent TB infection through the Isoniazid Preventive Therapy (IPT) intervention [10]. In epidemiological studies from different countries, IPT has been shown to reduce risks of TB incidence occurrence by 33–90% among HIV infected people [7, 10, 18–27]. WHO recommends the completion of at least 6 months IPT for the successful prevention of active tuberculosis among PLHIV [4, 10]. Globally, six-month IPT completion has been reported to range from 39 to 99% [28–37], and in Tanzania studies reported 65–98% of those who initiated IPT, completed the 6 month treatment course [6, 20, 38–40]. Most epidemiological studies have shown that IPT completion reduces the risks of mortality among PLHIV [8, 26, 41], decreases the need for expensive TB treatment needs and lowers the cost of HIV services. Successful use of IPT will help Tanzania achieve the sustainable development goals (SDG) by 2030 and is part of the national strategy to control TB in Tanzania [6, 7, 42, 43]. However, it shows that a good number of PLHIV do not complete the full six-month IPT treatment course and little is known about the determinants of IPT completion in routine HIV clinics settings. The iinformation on IPT completion would be useful to the Ministry of Health, Community Development, Gender, Elderly and Children (MOHCDGEC) in order to plan better interventions to prevent TB in among PLHIV [14, 20, 38, 44–46]. This study aimed to determine six-month IPT completion and factors associated with IPT completion among PLHIV aged 15 years or more in Dar es Salaam region, Tanzania.

## Methods

A cross-section study using secondary analysis of de-identified routinely collected data from PLHIV attending HIV services in 58 care and treatment clinics (CTC) was conducted in Dar es Salaam region. The study retrieved data from the CTC electronic database, which is used to record the clinical management of PLHIV attending CTC.

**Fig. 1:**
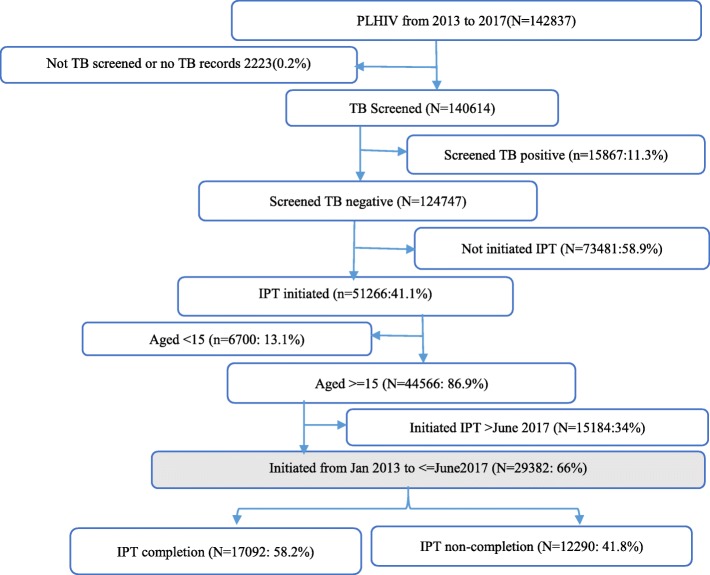
. Flow chart determining how participants were selected

The national TB/HIV and HIV guidelines recommend that all PLHIV should be screened for TB at every clinic visit using standard TB screening tools [4]. All PLHIV aged 15 years or more who screened negative for TB and who were initiated on IPT between January 2013 and June 2017 were included in the analysis (Fig. 1).

All subsequent clinic records of those who initiated IPT were reviewed for six-month IPT completion at their scheduled monthly visits. The outcome variable, six-month IPT completion, was defined as the collection of IPT drugs for the full 6 months following initiation. The independent variables were sex, age, functional status, pregnancy status, marital status, ART status, WHO clinical stage as well as CD4 count cells/μL at IPT initiation. The study used the records of all patients who initiated IPT in care and treatment centres (CTC) from all health facilities in Dar es Salaam region from January, 2013 to June, 2017. Figure 1 shows the total clients enrolled in HIV care, and the exclusion for different reasons (Fig. 1).

Data analysis was performed using STATA version 15.0 (STATA Corp, College station, TX). Descriptive statistics were summarized using means, standard deviation (SD)) and medians (IQR) for continuous variables; frequency and proportion for category variables. The chi-square test was used to compare the differences in the proportion of completing IPT across the patient characteristics. A modified Poisson regression model was used to determine the prevalence ratio (PR) and 95% confidence interval (95% CI) of factors associated with IPT completion. Multilevel modelling was used to estimate the independent determinants of IPT completion, while accounting for the dependency of individuals within health facilities. Intra class correlation was used to measure the proportion of variability explained by the between health facilities variation.

The study involved analysis of unlinked data; hence, there was no contact with human subjects. Ethics approval for the study was obtained from the Kilimanjaro Christian Medical University college - Research and Ethical Review Committee in 2018 (KCMUCo-RERC approval number 2388). Secondary use of the data from the electronic database was requested and approved by National Aids Control Program (NACP). NACP owns the data on behalf of MoHCDGEC. Patient consent was not required for the analysis of anonymised routine data.

## Results

A total of 142,837 PLHIV were registered in CTC in Dar-es-Salaam in the study period. Of these 2223 (0.2%) had no record of TB screening. Of 140,614 PLHIV, 124,747 (88.7%) had record of a negative TB screening results. Of those who screened negative 51,266 (41%) initiated IPT, but we excluded 6700 (13.1%) who were aged less than 15 years and 15,184 who initiated after 1st June 2017, leaving a total of 29,382 PLHIV in the analysis (Fig. 1).

The demographic and clinical characteristics of the 29,382 participants who initiated IPT are shown in Table 1 with 21,808 (74.2%) female, and a mean age of 41.4 years (standard deviation SD: 10.2) (Table 1.). The majority 28,859 (98.2%) were on ART, and the median CD4 counts was 155 cells/μL (interquartile range (IQR): 48–260), although a large group 13,687 (46.8%) had CD4 counts less than 100 cells/μL. Majority 28,480 (97.1%) PLHIV were actively attending clinics and 26,512 (90.3%) were attending in their scheduled appointment dates (Table 1).

**Table 1:** Demographic and Clinical characteristics of 29,382 Participants Attending HIV Services in Dar es Salaam Region

Variable	Frequency	%	IPT completers ***N*** = 17,092(58.2)	***P***-value
**Age category**				
15–24	1454	4.9	818(56.3)	
25–34	5216	17.8	2999(57.5)	
35–44	11,708	39.9	6771(57.8)	
≥45	11,004	37.4	6504(59.1)	
Mean (standard deviation)	41.4(±10.2)		41.55(±10.1)	0.053
**Sex**				
Male	7574	25.8	12,702(58.2)	
Female	21,808	74.2	4390(57.9)	0.667
**Marital status (*n*** **= 25,888)**				
Single	7847	30.3	4414(56.2)	
Married/cohabiting	13,401	51.8	7945(59.3)	
Divorced/widowed	4640	17.9	2823(60.8)	< 0.001
**Year of Initiation**				
2013	1857	6.3	773(41.6)	
2014	5169	17.6	2254(43.6)	
2015	7754	26.4	3783(48.8)	
2016	10,746	36.6	7353(68.4)	
2017 (Jan to June only)	3856	13.1	2929(75.9)	< 0.001
**PLHIV by Health facility ownership**				
Public	20,272	68.9	10,699(52.8)	
Private	9110	31.1	6393(70.2)	< 0.001
**ART status (*n*** **= 29,374)**				
Not on ART	515	1.8	136(26.4)	
On ART	28,859	98.2	16,956(58.7)	< 0.001
**WHO clinical stage (n = 29,121)**				
I	5026	17.3	3141(62.5)	
II	6758	23.2	4017(59.4)	
III	14,910	51.2	8505(57.1)	
IV	2427	8.3	1348(55.5)	< 0.001
**CD4 count in** cells/μL **(n = 29,249)**				
< 100	13,687	46.8	8001(58.5)	
100–349	9927	33.9	5999(60.4)	
≥ 350	5635	19.3	3016(53.5)	< 0.001
Median (lower quartile, upper quartile)	155(48, 260)			
**Visit status (n = 29,337)**				
Actively attending clinics	28,480	97.1	16,720(58.7)	
Missing scheduled appointments	383	1.3	186(48.6)	
Transferred to another clinic	474	1.6	174(36.7)	< 0.001
**Co-medications (*n*** **= 10,047)**				
Cotrimoxazole	7180	71.5	4160(57.9)	
Fluconazole	22	0.2	9(40.9)	
Others	2845	28.3	1743(61.3)	0.002
**Visit type (n = 29,354)**				
Scheduled	26,512	90.3	15,435(58.2)	
Traced back after LTFU	238	0.8	161(67.7)	
Treatment supported drugs pick up	287	1.0	174(60.6)	
Unscheduled	2317	7.9	1309(56.5)	0.007

**Fig. 2:**
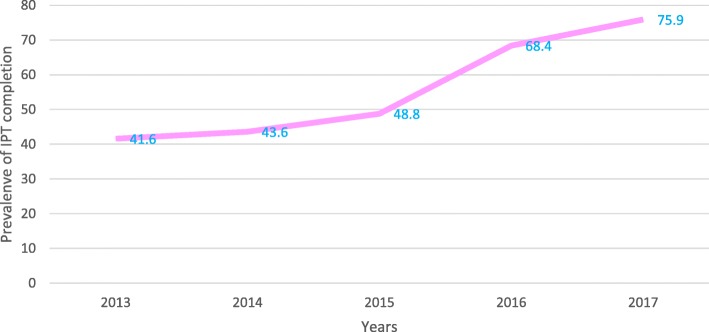
. Trend of IPT completion prevalence by years

Over the period January 2013 to December 2017, the overall IPT completion prevalence was 58%, but the IPT completion increased from 42% in 2013 to 76% in 2017 (Fig. 2).

Univariate analysis accounting for clustering at health facility level, indicated that the six-month IPT completion was 50% lower among those who were not on ART (PR: 0.50: 95%CI: 0.42–0.59) compared to those were on ART (Table 2). PLHIV who received IPT at private clinics had a 16% higher prevalence of IPT completion compared to those in public health facilities (PR: 1.16: 95%CI: 0.99–1.36) although this was statistically non-significant. PLHIV with WHO Stage III (PR: 1.01: 95%CI: 0.96–1.04) HIV disease had higher IPT completion compared to those with WHO Stage I. PLHIV with WHO Stage IV (PR: 0.99: 95%CI: 0.93–1.06) HIV disease lower six-month completion of IPT compared to those with WHO Stage I HIV disease. PLHIV with high CD4 counts of ≥350 cells/μL had a 6% lower IPT completion (PR: 0.94: 95%CI: 0.90–0.98) compared to those with CD4 count < 100 cells/μL.

**Table 2:** Determinants of IPT completion among PLHIV adjust for clustering at health facilities

Variable	CPR(95%CI)	ICC	***P***-value	APR(95%CI)	***P***-value	ICC
**Age category**						
15–24	0.97(0.90–1.05)	8.5%	0.439	0.97(0.89–1.04)	0.336	
25–34	0.94(0.89–0.98)		0.003	0.94(0.89–0.98)	0.010	
35–44	0.97(0.94–1.01)		0.091	0.97(0.94–1.01)	0.182	
≥ 45	Reference			Reference		
**Sex**						
Female	Reference			Reference		
Male	1.01(0.97–1.04)	8.5%	0.710	0.99(0.96–1.03)	0.876	
**Years of IPT**						
2013	Reference			Reference		
2014	0.93(0.85–1.02)		0.121	0.92(0.84–1.02)	0.101	
2015	0.98(0.89–1.08)		0.736	0.98(0.89–1.07)	0.621	
2016	1.26(1.16–1.38)		< 0.001	1.24(1.14–1.36)	< 0.001	
2017	1.37(1.25–1.50)	6.3%	< 0.001	1.34(1.22–1.47)	< 0.001	
**Marital status**						
Married/cohabiting	Reference			Reference		
Single	0.98(0.95–1.02)		0.384			
Divorced/widowed	1.01(0.96–1.05)	8.5%	0.944			
**Health facility ownership**						
Public	Reference			Reference		
Private	1.16(0.99–1.36)	7.9%	0.067	1.11(0.97–1.26)	0.144	
**ARV status**						
On ARV	Reference			Reference		
Not on ARV	0.50(0.42–0.59)	8.3%	< 0.001	0.54(0.45–0.64)	< 0.001	
**WHO stage**						
I	Reference			Reference		
II	0.98(0.94–1.03)		0.553	0.98(0.94–1.04)	0.613	
III	1.01(0.96–1.04)		0.986	0.98(0.94–1.02)	0.376	
IV	0.99(0.93–1.06)	8.4%	0.907	0.97(0.91–1.04)	0.455	
**CD4 counts in cells/*mm***^**3**^						
< 100	Reference			Reference		
100–349	0.96(0.93–0.99)		0.022	0.97(0.94–1.01)	0.083	
≥ 350	0.94(0.90–0.98)	8.5%	0.007	0.96(0.92–1.01)	0.085	
**Client visit status**						
Attending in the clinics at which client was initially enrolled in HIV care	Reference			Reference		
Missing appointments	0.87(0.76–1.01)		0.068	0.88(0.76–1.02)	0.098	
Transferred to another clinics within Dar es Salaam	0.63(0.60–0.71)	8.5%	< 0.001	0.63(0.54–0.74)	< 0.001	5.8%

*Key* CPR-Crude prevalence ratio, CI-Confidence interval and APR-Adjusted prevalence ratio; ICC: Intra cluster correlation

Other adjusted covariates are: functional status and visit type

Multivariate multilevel analysis then adjusted for all variables which were statistically significant at univariate analysis or had clinical meaning or were identified a priori as potential confounders. The variables which were adjusted for were age, sex, year of IPT initiation, ART status, clinical stage (important variable), CD4 counts in cells/μL and client visit status as well as accounting the random effects due to specific health facility. The adjusted PR shows patients not on ART had a 46% lower IPT completion when compared to those not on ART (APR: 0.54: 95%CI: 0.45–0.64). PLHIV who transferred to other clinics within Dar es Salaam region had 37% lower IPT completion compared to those were attending in the same clinics (APR:0.63: 95%CI:0.54–0.74). Compared to 2013 the prevalence of completing IPT was 24% higher in 2016 (APR: 1.24: 95%CI: 1.14–1.36) and 34% higher in 2017 (APR: 1.34: 95%: 1.22–1.47). The IPT completion rate was 6% lower among PLHIV aged 25–34 years compared to those aged 15 to 24 years (APR:0.94: 95%CI:0.89–0.98). The random effects results showed that 5.8% of the variability in IPT completion was due to differences between health facilities (Table 2).

## Discussion

These data show the completion of IPT among PLHIV who are attending routine HIV services in Dar-es-Salaam, Tanzania. The overall IPT completion was 58% and it increased over the 4 years from 42% in 2013 to 76% in 2017. This probably reflects the improved HIV services provided in the health facilities in Tanzania with better linkage between TB and HIV services, increased HIV testing and immediate ART initiation for those testing positive [47]. Since 2013 TB and HIV services have been devolved to lower level health facility in Tanzania [47].

Among the clients who did not complete IPT in the recommended period of 6 months, 96.2% discontinued IPT within the first 3 months of initiation. This is consistent with findings from Malawi, Zimbabwe and Ethiopia, which showed of those who stopped, 85, 89 and 89% respectively stopped in the first month [32, 48, 49]. Patients may be discouraged by the long treatment duration of IPT and the need to attend clinic every month [32, 48, 49]. Interventions have been considered to help new patients in those first few months, such as automated mobile phone short message services (SMS), smart-phone applications and home visits by lay counsellors as these have been shown to be effective in Malawi [49].

PLHIV who were transferred to another clinics within Dar es Salaam region after IPT initiation had significantly lower IPT completion as compared to those attended the same clinic for the whole 6 months. This result is similar to the findings from Zimbabwe and Ethiopia [27, 32]. The significantly higher IPT completion rate among PLHIV on ART is consistent with results obtained in Kenya, Zimbabwe and Brazil [30, 32, 37]. These results indicate that patients who are already linked with ART services have an increased prevalence of IPT completion. The significantly lower prevalence of IPT completion among those aged 25–34 years compared to those aged 15–24 years was consistent with a study in Uganda, but contrary to the results from Malawi [36, 49]. HIV patients with high CD4 counts ≥350 cells/μL had lower prevalence of IPT completion compared with those with CD4 counts < 100 cells/μL. This is consistent with studies in Kenya and Brazil [30, 37]. Possibly patients with high CD4 counts perceive themselves healthy and not at risk of progressing to active TB, so they stop taking IPT before completing the full treatment course of six months.

There was non-significant lower IPT completion among clients with advanced stage HIV disease, as shown by PLHIV with low CD4 counts, differed from findings from a study in Malawi [48]. PLHIV with advanced HIV stage are at higher risk of death which would be a reason for them not completing IPT although this was not statistically significant in a study in Zimbabwe [32]. PLHIV attending private health facilities had higher IPT completion compared to those attending public facilities althouhg this was not significant in this analysis. The differential might imply socio-economic inequities and difference in services costs between private and public health facilities, which was shown to influence IPT completion in Jamaica and Uganda [36, 50]. Although Tanzanian guidelines for HIV services cover both public and private facilities, more could be done to harmonize the practice styles and skills between private and public clinics.

The current study had strenghs due to large sample size and high power ≥ 90% to detect the true association between IPT completion and with its determinants. The study used routinely collected data, which reflected the real practices in Dar es Salaam, and the same analysis can be incorporated into regular analyses of the CTC data across Tanzania. The study included longer time interval which helped to account for population changes since IPT program was rollout in Tanzania. The study used modified Poisson regression, with robust standard errors, to avoid biases and to account for overdispersion. The study accounted for the effects of health facilities by using random effects to adjust for the clustering in the health facility. One limitation was the incompleteness and missing data which might have underestimated or overestimated the estimated prevalence ratios of IPT completion. Another limitation is that the study used routinely collected data which are collected for patient mangaement and programatic purposes, and not for research puporses. Therefore some important variables which are possibly associated with IPT completion are not routinely collected and recorded in the CTC2 database.

## Conclusions

IPT completion rate prevalence in Dar es Salaam region was low in 2013 but increased over time. This corresponds with a general improvement in HIV services, and the monitoring and evaluation of HIV services in Tanzania. It is important to maintain that increase to ensure people living with HIV are protected against TB disease. Targeted interventions need to be developed to assist patients with lower IPT completion. Further analysis of routinely collected health data should be done to monitor the changes in IPT completion among PLHIV. More research is needed on the effects of IPT non-completion rates and its reasons among PLHIV who initiate IPT in Tanzania.

## Data Availability

The data that support the findings of this study are available from the Ministry of Health Community Development Gender Elderly and Children (MOHCDGEC) but restrictions apply to the availability of these data, which were used under license for the current study, and so are not publicly available. Data are however available from the authors upon request and with permission from MOHCDGEC.

## Abbreviations

CPR: Crude prevalence ratio
APR: Adjusted prevalence ratio
ART: Antiretroviral therapy
CI: Confidence interval
CTC: Care and treatment clinics
RERC: Research and Ethical Review Committee
HIV: Human Immunodeficiency virus
IPT: Isoniazid preventive therapy
IQR: Inter quartile range
KCMUCO: Kilimanjaro Christian Medical University College
MOHCDGEC: Ministry of Health, Community Development, Gender Elderly and Children
PLHIV: People Living with HIV
TB: Tuberculosis
WHO: World Health Organization

## References

[1] WHO UNICEF and UNAIDS. A Guide on Indicators for Monitoring and Reporting on the Health Sector Response to HIV / AIDS [Internet]. 2011. Available from: http://www.who.int/hiv/pub/2010progressreport/report/en/.

[2] WHO. The global Tuberculosis report. [Internet]. 2014. Available from: http://www.who.int/tb/publications.

[3] Getachew Y, Mekonnen W. Correlates of adherence and utilization of isoniazid preventive therapy in HIV patients. Res Artic Infect Dis [Internet] 2015;5(2):45–50. Available from: www.jmidonline.org.

[4] MOHCDGEC. National Guidelines for Management of HIV and AIDS Updated 11July 2017 to incoprporate BAKITA &CDC 11.07.2017. Dar es Salaam; 2017.

[5] MOHCDGEC. National opperational guideline for community based TB, TB/HIV and DR-TB interventions. Dar es Salaam: NTLP; 2016.

[6] Munseri PJ, Talbot EA, Mtei L, von Reyn CF (2008). Completion of isoniazid preventive therapy among HIV-infected patients in Tanzania. Int J Tuberc Lung Dis.

[7] WHO. Global tuberculosis Report 2017 [Internet]. 2017. Available from: http://www.who.int/tb/publications.

[8] Floyd K, Glaziou P, Zumla A, Raviglione M. The global tuberculosis epidemic and progress in care, prevention, and research: an overview in year 3 of the End TB era. Lancet Respir Med 2018. 2018;6:299–314. 10.1016/S2213-2600(18)30057-2 29595511

[9] Gunda DW, Maganga SC, Nkandala I, Kilonzo SB, Mpondo BC, Shao ER, et al. Prevalence and risk factors of active TB among adult HIV patients receiving ART in northwestern Tanzania: a retrospective cohort study. 2018;. 10.1155/2018/1346104 PMC6057398 30073038

[10] WHO. Global Tuberculosis Report; World Health Orgarnization (WHO) [Internet]. 2018. Available from: http://www.who.int/tb/joint-initiative/en/.

[11] NTLLP. Kenya National Strategic Plan on Tuberculosis, Leprosy and Lung Health 2015–2018 [Internet]. 2014. Available from: http://healthservices.uonbi.ac.ke/sites/default/files/centraladmin/healthservices/Kenya National Strategic Plan on Tuberculosis, Leprosy.pdf.

[12] WHO. Use of high burden country lists for TB by WHO in the post-2015 era [Internet]. Geneva; 2015. Available from: http://whqlibdoc.who.int/hq/1998/WHO_TB_98.245.pdf?ua.

[13] COP. Country operational plan (COP) strategic direction summary. 2016.

[14] NTLP. National tuberculosis and leprosy programe in Tanzania [internet]. Dar Es Salaam; 2006. Available from: www.ntlp.go.tz.

[15] Maokola W, Ngowi B, Lawson L, Mahande M, Todd J, Msuya S. Performance of and factors associated with TB screening and diagnosis using Sputum microscopy examination among People Living with HIV: analysis of 2012–2016 routine HIV data in Tanzania. Front Public Heal. 2020;7(404). 10.3389/fpubh.2019.00404 PMC7015871 32117844

[16] Zhao Y, Li M, Yuan S. Analysis of Transmission and Control of Tuberculosis in Mainland China, 2005–2016, Based on the Age-Structure Mathematical Model. Int J Environ Res Public Health. 2016:535–9. 10.3390/ijerph14101192 PMC5664693 28991169

[17] CDC. Isoniazid (INH) for the Treatment of TB Infection NOTE: United State of America, Califonia; 2017. p. 1–2.

[18] Kahwati LC, Feltner C, Halpern M, Woodell CL, Boland E, Amick HR, et al. Screening for Latent Tuberculosis Infection in Adults: An Evidence Review for the U.S. Preventive Services Task Force. Agency Healthc Res Qual. 2016;142(14–05212-EF-1 September). 27656733

[19] Ayele AA, Atnafie SA, Balcha DD, Weredekal AT, Woldegiorgis BA, Wotte MM, et al. Self-reported adherence and associated factors to isoniazid preventive therapy for latent tuberculosis among people living with HIV/AIDS at health centers in Gondar town. North West Ethiop. 2017:743–9. 10.2147/PPA.S131314 PMC5391840 28435232

[20] Sabasaba A, Mwambi H, Somi G, Ramadhani A, Mahande J M. Effect of isoniazid preventive therapy on tuberculosis incidence and associated risk factors among HIV infected adults in Tanzania:a retrospective cohort study. BMC Infect Dis. 2019;2019:19–62. Available from: https://doi.org/10.1186/s12879-019-3696-x%0A(2019). 10.1186/s12879-019-3696-x PMC6337848 30654753

[21] Getahuna H, Granichb R, Sculiera D, Gunneberga C, Blanca L, Nunna P, et al. Implementation of isoniazid preventive therapy for people living with HIV worldwide: barriers and solutions. AIDS. 2010;24 (suppl(2010):S57–S65. Available from: ISSN 0269–9370 Q 2010 Wolters Kluwer Health %7C Lippincott Williams & Wilkins Copyright © Lippincott Williams & Wilkins. 10.1097/01.aids.0000391023.03037.1f 21079430

[22] Granich R, Akolo C, Gunneberg C, Getahun H, Williams P, Williams B. Prevention of tuberculosis in people living with HIV [internet]. Geneva: Clinical Infectious Diseases; 2010. Available from: http://cid.oxfordjournals.org/. 10.1086/651494 20397951

[23] Falzon D, Getahun H, Kanchar A, Mirzayev F, Raviglione M, Timimi H, et al.. Use of high burden country lists for TB by WHO in the post-2015 era. 2015;(April).

[24] Alsdurf H, Hill PC, Matteelli A, Getahun H, Menzies D. The cascade of care in diagnosis and treatment of latent tuberculosis infection: a systematic review and meta-analysis. 2016;16(30216–X):S1473–3099. 10.1016/S1473-3099(16)30216-X 27522233

[25] Hart L, Hamilton C, Boocher K. Isoniazid preventive therapy for the prevention of tuberculosis in people living with HIV / AIDS providing isoniazid to. 2011.

[26] Samandari T, Agizew TB, Nyirenda S, Tedla Z, Sibanda T, Mosimaneotsile B, et al. Tuberculosis incidence after 36 months’ isoniazid prophylaxis in HIV-infected adults in Botswana: a posttrial observational analysis. PMC 2016 December 01. 2015;28; 29(3): 351–359. 10.1097/QAD.0000000000000535 PMC5131631 25686683

[27] Ayele HT, van Mourik MS, Bonten MJ (2015). Effect of isoniazid preventive therapy on tuberculosis or death in persons with HIV: a retrospective cohort study. BMC Infect Dis.

[28] Al-Darraji HAA, Kamarulzaman A, Altice FL. Isoniazid preventive therapy in correctional facilities: a systematic review. Int J Tuberc Lung Dis 2012;16(7):871–879. Available from: http://dx.doi.org/10.5588/ijtld.11.0447. 10.5588/ijtld.11.0447 22410101

[29] Ousley J, Soe KP, Kyaw NTT, Anicete R, Mon PE, Lwin H, et al. IPT during HIV treatment in Myanmar: high rates of coverage, completion and drug adherence. Public Heal Action. 2018;8(1). 10.5588/pha.17.0087 PMC5858061 29581939

[30] Giselle I, Bonnie SK, Cohne S, Efron A, Antonio GP, Lawrence HM (2010). The implementation of isoniazid preventive therapy in HIV clinics: the experience from the TB/HIV in Rio (THRio) study. NIH Public Access.

[31] Dhungana GP, Thekkur P, Chinnakali P, Bhatta U, Pandey B, Zhang W-H (2019). Open access research initiation and completion rates of isoniazid preventive therapy among people living with HIV in far-Western region of Nepal: a retrospective cohort study. BMJ Open Access.

[32] Takarinda KC, Choto RC, Harries AD, Mutasa-Apollo T, Chakanyuka-Musanhu C. Routine implementation of isoniazid preventive therapy in HIV-infected patients in seven pilot sites in Zimbabwe. Public Heal Action [Internet]. 2017; Available from: www.theunion.org. 10.5588/pha.16.0102 PMC5526481 28775944

[33] Maharaj B, Gengiah TN, Nonhlanhla Y-Z, Gengiah S, Naidoo A, Naidoo K. Implementing Isoniazid Preventive Therapy in a TB-treatment experienced cohort on ART. Vol. 4. 2017. 10.5588/ijtld.16.0775 PMC5696541 28399969

[34] Oni T, Tsekela R, Kwaza B, Manjezi L, Bangani N, Wilkinson KA, et al. A Recent HIV Diagnosis Is Associated with Non- Completion of Isoniazid Preventive Therapy in an HIV- Infected Cohort in Cape Town. PLoS One. 2012;7(12):e52489. Available from: www.plosone.org. 10.1371/journal.pone.0052489 PMC3527556 23285064

[35] Yotebieng M, Edmonds A, Lelo P, Wenzi LK, Bu PTN-, Lusiama J, et al. High completion of isoniazid preventive therapy among HIV-infected children and adults in Kinshasa, Democratic Republic of Congo. Res Lett AIDS 2015. 2015;29:2055–2060. 10.1097/QAD.0000000000000791 PMC4566165 26352882

[36] Tram KH, Mwangwa F, Atukunda M, Owaraganise A, Ayieko J, Plenty A, et al. Isoniazid Preventive Therapy Completion in the Era of Differentiated HIV Care. J Acquir Immune Defic Syndr. 2017;76(5). Available from: Copyright © 2017 The author(s). Published by Wolters Kluwer Health, Inc. www.jaids.com. 10.1097/QAI.0000000000001540 PMC5680115 28885271

[37] LaCourse M S, Graham M S, Jacko W, Deya W R, Masese N L, Mandaliya N K, et al. Evaluation of the isoniazid preventive therapy care Casecade among HIV-positive female sex Workers in Mombasa, Kenya. JAIDS J Acquir Immune Defic Syndr Publ Ahead Print DOI 2014;. 10.1097/QAI.0000000000001461 PMC5555166 28797022

[38] Shayo GA, Moshiro C, Aboud S, Bakari M, Mugusi FM. Acceptability and adherence to Isoniazid preventive therapy in HIV-infected patients clinically screened for latent tuberculosis in Dar es Salaam, Tanzania. BMC Infect Dis. 2015;2015(15):368. Available from: www.biomedcentral.com/submit. 10.1186/s12879-015-1085-7 PMC4549887 26306511

[39] Kabali C, von Reyn CF, Brooks DR, Waddell R, Mtei L, Bakari M (2011). Completion of isoniazid preventive therapy and survival in HIV-infected, TST-positive adults in Tanzania. Int J Tuberc Lung Dis.

[40] Bakari M, Sandstrom E, Mhalu F, Pallangyo K. Isoniazid prophylaxis for tuberculosis prevention among HIV infected police officers in Dar Es Salaam. East Africa Med J. 2000. 10.4314/eamj.v77i9.46695 12862141

[41] Glaziou P, Floyd K, Sismanidis C, Raviglione M. Global Epidemiology of Tuberculosis. Cold Spring Harb Perspect Med [Internet]. 2015; Available from: www.who.int/tb/data. 10.1101/cshperspect.a017798 PMC4315920 25359550

[42] WHO. Guidelines for managing advanced HIV disease and rapid initiation of antiretroviral therapy. Behav Inf Technol. 2017 Apr 7;2(2):127–161. Available from: http://apps.who.int/iris/bitstream/10665/255884/1/9789241550062-eng.pdf?ua=1. 29341560

[43] UNAIDS, WHO, CDC, PEPFAR, FHi360. Global HIV Strategic Information Working Group Survey Guidelines Biobehavioural:For Populations At Risk For HIV 2017.

[44] Bakari M, Mhalu F, Sandstrom E, Pallangyo K. Isoniazid prophylaxis for tuberculosis prevention among HIV infected police officers in Dar Es Salaam. East Africa Med J. 2000;2000:494–497. Available from: https://www.researchgate.net/publication/10660678%0AIsoniazid. 10.4314/eamj.v77i9.46695 12862141

[45] Said K, Hella J, Mhalu G, Chiryankubi M, Masika E, Maroa T, et al. Diagnostic delay and associated factors among patients with pulmonary tuberculosis in Dar es Salaam, Tanzania Khadija. Res Artic Infect Dis poverty. 2017; Available from: http://creativecommons.org/publicdomain/. 10.1186/s40249-017-0276-4 PMC5364704 28335816

[46] Nagu TJ, Aboud S, Matee MI, Maeurer MJ, Fawzi WW, Mugusi F. Effects of isoniazid resistance on TB treatment outcomes under programmatic conditions in a high-TB and -HIV setting: a prospective multicentre study. J Antimicrob Chemother Adv access Publ December 20, 2016 J Antimicrob Chemothe 2016;. 10.1093/jac/dkw503 27999054

[47] UNAIDS. HIV and AIDS in Tanzania [Internet]. Tanzania; 2019. Available from: www.avert.org.

[48] Tadesse Y, Gebre N, Daba S, Gashu Z, Habte D, Hiruy N, et al. Uptake of isoniazid preventive therapy among under-five children: TB contact investigation as an entry point. PLos. 2016;. 10.1371/journal.pone.0155525 PMC4873181 27196627

[49] Thindwa D, MacPherson P, Choko AT, Khundi M, R Sambakunsi, Ngwira LG, et al. Completion of isoniazid preventive therapy among human immunodeficiency virus positive adults in urban Malawi. Int J Tuberc Lung Dis 2018;22(3):273–279. Available from: http://dx.doi.org/10.5588/ijtld.17.0370%250. 10.5588/ijtld.17.0370 PMC5824849 29471904

[50] Bourne PA, Eldemire-shearer D, Paul TJ, Lagrenade J, Charles Christopher a. Public and private health care utilization differences between socioeconomic strata in Jamaica Patient Relat Outcome Meas 2010;2010:81–91. 10.2147/PROM.S11868 PMC3417901 22915955

